# Microglial phagocytosis and activation underlying photoreceptor degeneration is regulated by CX3CL1‐CX3CR1 signaling in a mouse model of retinitis pigmentosa

**DOI:** 10.1002/glia.23016

**Published:** 2016-06-17

**Authors:** Matthew K. Zabel, Lian Zhao, Yikui Zhang, Shaimar R. Gonzalez, Wenxin Ma, Xu Wang, Robert N. Fariss, Wai T. Wong

**Affiliations:** ^1^ Unit on Neuron‐Glia Interactions in Retinal Disease National Eye Institute, National Institutes of Health Bethesda Maryland; ^2^ Biological Imaging Core National Eye Institute, National Institutes of Health Bethesda Maryland

**Keywords:** retina, inflammation, apoptosis, chemokine, neuroprotection

## Abstract

Retinitis pigmentosa (RP), a disease characterized by the progressive degeneration of mutation‐bearing photoreceptors, is a significant cause of incurable blindness in the young worldwide. Recent studies have found that activated retinal microglia contribute to photoreceptor demise via phagocytosis and proinflammatory factor production, however mechanisms regulating these contributions are not well‐defined. In this study, we investigate the role of CX3CR1, a microglia‐specific receptor, in regulating microglia‐mediated degeneration using the well‐established rd10 mouse model of RP. We found that in CX3CR1‐deficient (CX3CR1^GFP/GFP^) rd10 mice microglial infiltration into the photoreceptor layer was significantly augmented and associated with accelerated photoreceptor apoptosis and atrophy compared with CX3CR1‐sufficient (CX3CR1^GFP/+^) rd10 littermates. CX3CR1‐deficient microglia demonstrated increased phagocytosis as evidenced by (1) having increased numbers of phagosomes *in vivo*, (2) an increased rate of phagocytosis of fluorescent beads and photoreceptor cellular debris *in vitro*, and (3) increased photoreceptor phagocytosis dynamics on live cell imaging in retinal explants, indicating that CX3CR1 signaling in microglia regulates the phagocytic clearance of at‐risk photoreceptors. We also found that CX3CR1 deficiency in retinal microglia was associated with increased expression of inflammatory cytokines and microglial activation markers. Significantly, increasing CX3CL1‐CX3CR1 signaling in the rd10 retina via exogenous intravitreal delivery of recombinant CX3CL1 was effective in (1) decreasing microglial infiltration, phagocytosis and activation, and (2) improving structural and functional features of photoreceptor degeneration. These results indicate that CX3CL1‐CX3CR1 signaling is a molecular mechanism capable of modulating microglial‐mediated degeneration and represents a potential molecular target in therapeutic approaches to RP. GLIA 2016;64:1479–1491

## Introduction

RP comprises a category of inherited retinal diseases in which mutations in photoreceptor or retinal pigment epithelium genes result in progressive degeneration of photoreceptors (Hartong et al., 2006). Affected patients, who bear mutations in one of a large (>200) set of causative genes (Daiger et al., 2013), progress typically to severe vision loss. Currently, RP lacks comprehensive treatment (Sacchetti et al., 2015) and is a leading cause of blindness in the young worldwide (Bunker et al., 1984; Gao et al., 2015; Haim, 2002). Elucidating cellular and molecular mechanisms common to various genetic etiologies of RP is of key importance in the search for treatments that can slow disease progression and vision loss (Sahel et al., 2015).

While cell‐autonomous mechanisms within mutation‐bearing photoreceptors are central to disease initiation (Sancho‐Pelluz et al., 2008), recent studies have highlighted the contributions of non‐cell‐autonomous mechanisms involving inflammation (Mustafi et al., 2012; Yoshida et al., 2013), particularly with respect to retinal microglia (Gupta et al., 2003; Roque et al., 1996). We have recently discovered that retinal microglia in RP mouse models and human RP contribute directly to photoreceptor degeneration via non‐cell‐autonomous mechanisms involving primary phagocytosis and inflammatory cytokine production (Zhao et al., 2015). We found that activated retinal microglia at the early onset of degeneration infiltrate photoreceptor layer and interact closely with mutation‐bearing rod photoreceptors via dynamic processes. These repeated contacts culminate eventually in the phagocytosis and removal of living rods, contributing to photoreceptor demise. Our current research goal is to discover molecular mechanisms regulating these microglia‐mediated processes so as to control and modulate the contributions of microglia to photoreceptor loss in RP.

CX3CR1 is a chemokine receptor that is specifically and constitutively expressed by microglia in the healthy developing and adult CNS (Ginhoux et al., 2010; Mizutani et al., 2012), including the retina (Carter and Dick, 2004; Combadiere et al., 2007) Its sole ligand, CX3CL1 (or fractalkine), expressed by CNS neurons either as a membrane‐bound or a secreted ligand (Harrison et al., 1998; Kim et al., 2011), prominently regulates neuron‐to‐microglia communication, modulating various aspects of microglial physiology, including migration, motility, and activation status (Arnoux and Audinat, 2015; Paolicelli et al., 2014). How CX3CR1 signaling results in neuroprotective vs. neurotoxic effects in different pathological contexts however remains incompletely elucidated (Lauro et al., 2015). In the retina, the loss of CX3CR1 signaling in retinal disease has been associated with alterations in pathological phenotypes in mouse models of retinal diseases such as diabetic retinopathy (Cardona et al., 2015; Kezic et al., 2013), glaucoma (Wang et al., 2014), uveitis (Dagkalis et al., 2009), oxidative injury (Chen et al., 2013), and light‐induced injury (Sennlaub et al., 2013). In particular, in the rd10 model of RP, CX3CR1 deficiency has also been associated with increased photoreceptor degeneration (Peng et al., 2014), but how this alteration results in changes in microglia‐photoreceptor interactions responsible for altered photoreceptor degeneration is not clear.

In the current study, we investigated how CX3CL1‐CX3CR1 signaling influences microglia‐photoreceptor interactions in the well‐characterized rd10 mouse model of RP (Chang et al., 2007) where a point mutation in the rod photoreceptor‐specific *Pde6b* gene, which is also causative in human RP (McLaughlin et al., 1993), results in progressive photoreceptor degeneration. The results obtained here indicate that CX3CL1‐CX3CR1 signaling regulates microglial phagocytosis and activation in this pathologic context, and in doing so, impacts the timing and tempo of photoreceptor degeneration. Modulation of this axis of signaling can constitute a potential molecular strategy to reduce microglial contributions to photoreceptor degeneration in RP which may serve to defer the onset and progression of vision loss in affected patients.

## Methods and Materials

### Experimental Animals

Experiments were conducted according to protocols approved by a local Institutional Animal Care and Use Committee and adhered to the Association for Research in Vision and Ophthalmology (ARVO) Statement for animal use in ophthalmic and vision research. Mice homozygous for the *Pde6b^rd10^* loss‐of‐function point mutation (rd10; Stock No. 004297) and for the loss‐of‐function CX3CR1‐GFP targeted mutation (CX3CR1^GFP/GFP^; Stock No. 005582) were obtained from The Jackson Laboratory (Bar Harbor, ME). Animals were genotyped and confirmed to lack the rd8 mutation (Mattapallil et al., 2012). Transgenic mouse lines were crossed together to generate *Pde6b^rd10/rd10^*, CX3CR1^GFP/GFP^ mice; these were subsequently crossed with *Pde6b^rd10/rd10^*, CX3CR1^+/GFP^ to generate *Pde6b^rd10/rd10^*, CX3CR1^GFP/GFP^, and *Pde6b^rd10/rd10^*, CX3CR1^+/GFP^ littermates (hereafter referred to as rd10;CX3CR1^GFP/GFP^ and rd10;CX3CR1^GFP/+^ respectively). Experiments involved animals in the range of ages from postnatal day (P)15‐P28 that were of mixed gender. Animals were housed in a National Institutes of Health animal facility under a 12‐h light/dark cycle with food *ad libitum*.

### Immunohistochemistry and TUNEL Labeling of Retinal Sections

Mice euthanized by carbon dioxide inhalation were enucleated. The resulting eyecups were marked for orientation and then placed in 4% paraformaldehyde (1 h at room temperature) for fixation and then embedded in 7% agarose. Retinal sections of 100 μm thickness that traversed the optic nerve in the superior‐inferior plane were prepared using a vibratome (VT1000, Leica). Sections were blocked and permeabilized (in 1xPBS, with 5% normal goat serum, 0.5% Triton X‐100 for 3 h at room temperature), and then incubated in primary antibodies in 1x PBS with 0.5% Triton X‐100 for 36 h at 4°C. Primary antibodies included rabbit anti‐Iba1 (Wako, #019‐19741, 1:500), rat anti‐CD68 (AbD Serotec, 1:500), mouse anti‐IL1β (Cell Signaling, #12242S, 1:50), and mouse anti‐CX3CL1 (R&D Systems, #AF537, 1:100). Sections were washed and then incubated overnight with secondary antibodies (Alexa Fluor‐488‐conjugated goat anti‐rabbit IgG for Iba1; Alexa Fluor‐568‐conjugated goat anti‐rabbit or rat IgG for CX3CL1 or CD68, respectively) and DAPI (1:500; Sigma). Experiments in which primary antibodies were omitted served as negative controls. Apoptotic photoreceptors were labeled with a terminal deoxynucleotidyl transferase dUTP Nick End Labeling (TUNEL) assay (Roche, Indianapolis, IN) according to the manufacturer's specifications. Stained retinal sections were imaged with confocal microscopy (FluoView 1000, Olympus). Multiplane z‐series were collected using a 40× oil‐immersion objective and analyzed with FV100 Viewer Software (Olympus) and Image J (NIH).

### In Vitro Phagocytosis Assays

Retinal microglia were cultured from CX3CR1^GFP/GFP^ and CX3CR1^GFP/+^ retinas as previously described (Ma et al. 2013) and seeded into six‐well plates (5 × 10^5^ cells/well). In one phagocytosis assay, cultured microglia were incubated with fluorescent bioparticles (1 mg mL^−1^, pHrodo Red *E. coli* Bioparticles®, #P35361, Life Technologies) for 2 h at 37°C according to the manufacturer's instructions. In a separate assay, photoreceptor debris was prepared by trypsinization and sonication of cultured 661W photoreceptor cells (gift of Dr. Muyyad Al‐Ubaidi, University of Oklahoma Health Sciences Center) that had been previously labeled with the lipophilic dye DiI (CellTracker™ CM‐DiI Dye, #C‐7001, Thermo Fisher Scientific). This was added to microglia cultures for 12 and 24 h to allow phagocytic uptake. Microglial phagocytosis of bioparticles or debris was imaged on an epifluorescence microscope.

### Live Time‐lapse Confocal Imaging

Microglial dynamic phagocytosis was examined by live‐cell time‐lapse confocal imaging in retinal explants from rd10;CX3CR1^GFP/GFP^ and rd10;CX3CR1^GFP/+^ mice at P22‐24 as previously described (Zhao et al., 2015). Briefly, retina explants were incubated in an oxygenated chamber in Ringer's solution containing propidium iodide (PI; 1:5000; Life Technologies) to label the nuclei of permeabilized cells and Hoechst 33342 (1:500; Life Technologies) to label all nuclei, before transfer to a temperature‐controlled (32°C) stage (Bioptechs) through which oxygenated Ringer's solution was superfused. Dynamic microglial behavior was followed with time‐lapse confocal imaging (FV1000, Olympus) using a 40× immersion objective. Z‐series stacks of microglia within the outer nuclear layer (ONL) were captured at a resolution of 1024 × 1024 pixels every 49 s for up to 2 h.

### Image Analysis

Morphological analyses were performed in retinal sections in the region of the inferior mid‐peripheral retina (0.75–1.25 mm radial distance from the optic nerve). Counts of retinal microglia, microglial phagosomes, and TUNEL+ nuclei in the ONL, were performed on z‐projections of confocal stacks of uniform depth. Mean thickness measurements of the ONL were computed across a 40× imaging field. For *in vitro* phagocytosis assays, areas of fluorescence (corresponding to intracellular fluorescent beads or DiI‐labelled 661W cell debris) were derived by thresholding images captured under uniform imaging conditions, and expressed as a fraction of the area covered by microglial cells. In time‐lapse imaging experiments, images were processed using ImageJ as previously described (Damani et al., 2011; Fontainhas et al., 2011). All GFP‐labeled microglia in the imaging field were scored for phagocytic events, including engulfment of photoreceptor nuclei and the subsequent development of PI‐labeling of engulfed nuclei; the mean rate of events were expressed as mean number of events per microglia per unit time. The proportion of PI+ nuclei in the ONL nuclei was also counted and computed for each recording field.

### Measurement of Cytokine Levels

Dissected mouse retinas were placed into 150 μL of protein lysate buffer (Complete Ultra, Roche) with proteinase inhibitor cocktail (Calbiochem, Gibbstown, NJ) at 4°C. Following sonication and centrifugation, protein concentration was measured (BCA protein assay kit, Pierce). Cytokine levels were determined using a Milliplex^®^ assay kit (Milliplex MAP mouse cytokine/chemokine magnetic bead panel, #MCYTOMAG‐70K, Millipore Corp) using the Luminex MAPIX system with data analysis using xPONENT 4.2 software (Luminex Corporation).

### Inhibition of CX3CR1 Signaling with Recombinant CX3CL1

To augment CX3CR1 signaling in microglia, exogenous full‐length recombinant mouse CX3CL1 (R&D Systems, #472‐FF/CF) was injected intravitreally into one eye of P20 rd10;CX3CR1^+/GFP^ mice (1 μL injection of 66–100 ng μL^−1^ solution, final vitreous concentration of 17.5–25 ng μL^−1^ in PBS). The contralateral control eye received an equal dose of heat‐inactivated CX3CL1 in PBS. Animals were sacrificed at P26 and their retinas analyzed. The order of eye (right or left) receiving CX3CL1 vs. control was alternated in experimental replicates.

### Electroretinographic Analysis

Electroretinographs (ERGs) were recorded in rd10 mice using an Espion E^2^ system (Diagnosys). Mice were dark adapted overnight, anesthetized with intraperitoneal ketamine (90 mg kg^−1^) and xylazine (8 mg kg^−1^), and dilated with topical tropicamide (1%, Alcon) and phenylephrine (2.5%, Alcon). Flash ERGs recordings were obtained simultaneously from both eyes with gold wire loop electrodes, with the reference electrode was placed in the mouth and the ground subdermal electrode at the tail. ERG responses were obtained at increasing light intensities over the ranges of 1 × 10^−4^−10 cd·s m^−2^ under dark‐adapted conditions, and 0.3–100 cd·s m^−2^ under a background light that saturates rod function. The stimulus interval between flashes ranged from 5 to 60 s (from lowest to highest stimulus strengths). ERG signals were sampled at 1 kHz and recorded with 0.3 Hz low‐frequency and 300 Hz high‐frequency cutoffs. Analysis of a‐wave and b‐wave amplitudes was performed using customized Espion ERG Data Analyzer software (v2.2).

### Statistical Analysis

All data were analyzed using statistical software (Graphpad Software). A normality test (D'Agostino and Pearson) was used to analyze the distribution of all data sets. For two‐way comparisons of data following a Gaussian distribution, independent data sets were analyzed with an unpaired two‐tailed *t* test; data not following a Gaussian distribution was analyzed with a non‐parametric Mann–Whitney test. Paired data sets were analyzed with a paired *t* test. For comparisons determining the effect of genotype on experimental animals of different ages, a two‐way ANOVA was employed. A *P* value < 0.05 was set as the basis for rejecting the null hypothesis.

## Results

### CX3CR1‐deficient Microglia Are Associated with Early and Augmented Photoreceptor Degeneration

**Figure 1:**
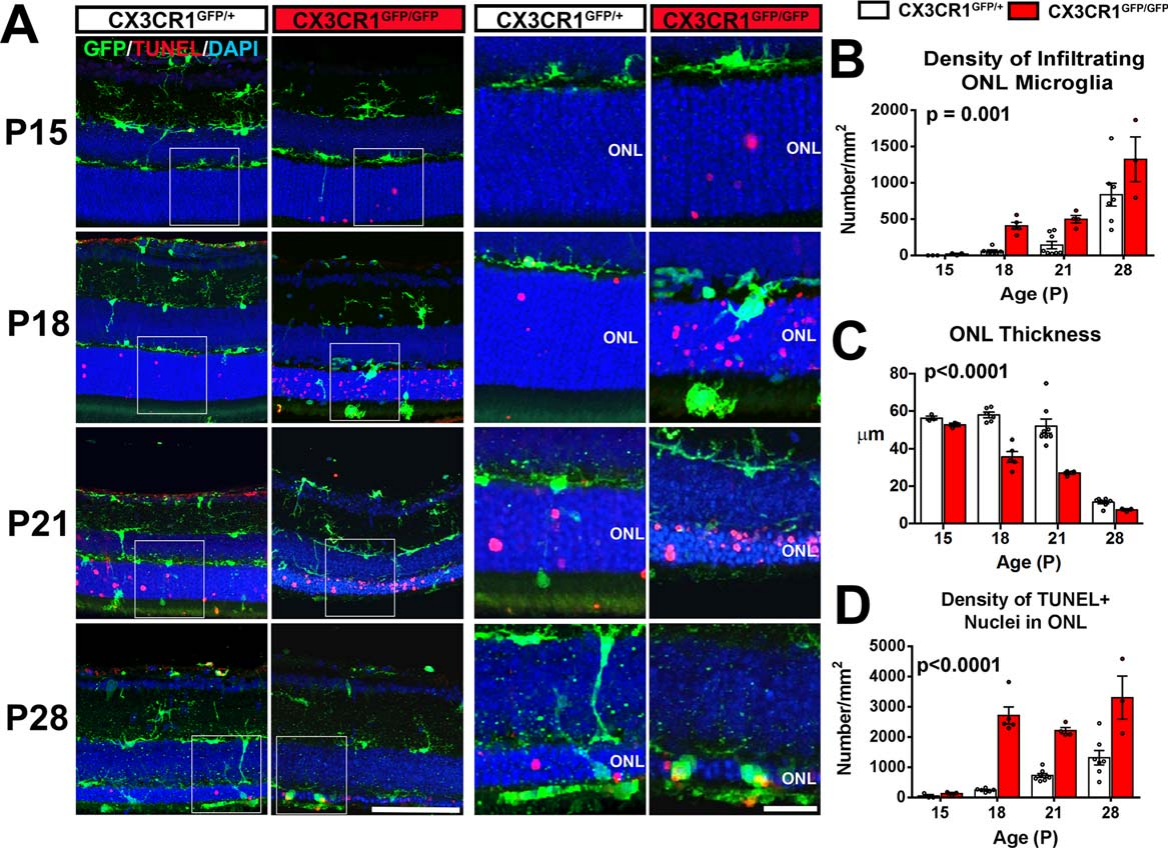
CX3CR1 deficiency is associated with early and augmented microglial infiltration into the outer nuclear layer (ONL) and with accelerated photoreceptor degeneration in the rd10 model of retinitis pigmentosa. (**A**) Comparison of retinal sections from rd10;CX3CR1^GFP/+^ and rd10;CX3CR1^GFP/GFP^ mice at postnatal (P) ages P15, P18, P21, and P28; panels (*right*) show magnified views of the ONL in insets (*left*). CX3CR1‐deficient microglia (*green*) in rd10;CX3CR1^GFP/GFP^ retina, relative to those in rd10;CX3CR1^GFP/+^ retina, infiltrated the ONL at earlier time‐points beginning at P18, with a concurrent emergence of TUNEL‐positivity in ONL nuclei (*red*). Scale bar, 50 μm; 20 μm for magnified views in insets. The density of infiltrating microglia in the ONL was greater in the rd10;CX3CR1^GFP/GFP^ retina at time‐points across the period of rod degeneration (**B**). Increased microglial infiltration was correlated with earlier and more extensive atrophy of the ONL (**C**) and a higher density of apoptotic TUNEL+ photoreceptors (**D**) in the rd10;CX3CR1^GFP/GFP^ retina. Column heights (in B, C, D) indicate mean, error bars indicate ±SEM, individual data points represent data from a single experimental animal (*N* = 3–8 animals of each genotype and age, *P* values indicate comparisons between genotypes across all time‐points, two‐way ANOVA). [Color figure can be viewed in the online issue, which is available at wileyonlinelibrary.com.]

To confirm and elucidate the role of retinal microglia in photoreceptor degeneration(Zhao et al., 2015), we investigated how CX3CR1 deficiency, a key regulator of microglial physiology (Combadiere et al., 2007; Liang et al., 2009), influences microglial involvement and disease progression in the rd10 model for RP. We compared CX3CR1‐sufficient rd10;CX3CR1^GFP/+^ animals with CX3CR1‐deficient, rd10;CX3CR1^GFP/GFP^ littermates prior to and during rod degeneration from P15‐P28 (Fig. 1A–D). We observed that at P15, the overall thickness of the ONL and the distribution of microglia in the inner retina was similar between genotypes. However, isolated TUNEL‐labeled apoptotic photoreceptor nuclei were detected in the ONL of rd10;CX3CR1^GFP/GFP^ animals but not in rd10;CX3CR1^GFP/+^ littermates. At P18, microglia in rd10;CX3CR1^GFP/GFP^ retinas infiltrated prominently into the ONL, interacting closely with photoreceptor nuclei, contrasting with microglia in rd10;CX3CR1^GFP/+^ retina which remained largely in the inner retina. At P21 and P28, microglia infiltration into the ONL was detected in both genotypes but infiltrating microglia numbers were significantly greater in rd10;CX3CR1^GFP/GFP^ retinas (Fig. 1B, *P* = 0.001, two‐way ANOVA). These observations indicated that CX3CR1‐deficient microglia may be more sensitive to presence of pathological stress in mutation‐bearing rods, and infiltrate the ONL earlier and in greater numbers relative to CX3CR1‐sufficient microglia.

These differences were temporally associated with an earlier and more rapid course of photoreceptor degeneration in rd10;CX3CR1^GFP/GFP^ retinas. These were evident as (1) an earlier onset of detectable ONL thinning commencing at P18 (compared with P21 in the rd10;CX3CR1^GFP/+^ retina) and (2) significantly reduced ONL thickness relative to the rd10;CX3CR1^GFP/+^ retina (Fig. 1C, *P* < 0.0001, two‐way ANOVA). The density of apoptotic TUNEL+ nuclei in the ONL across the entire period was also greater in rd10;CX3CR1^GFP/GFP^ retinas (Fig. 1D, *P* < 0.0001, two‐way ANOVA). These findings indicate that changes in retinal microglia conferred by the absence of CX3CR1 are capable of contributing in non‐cell autonomously to photoreceptor degeneration progression in RP.

### CX3CR1‐Deficient Microglia Demonstrate Increased Phagocytosis of Photoreceptors

**Figure 2:**
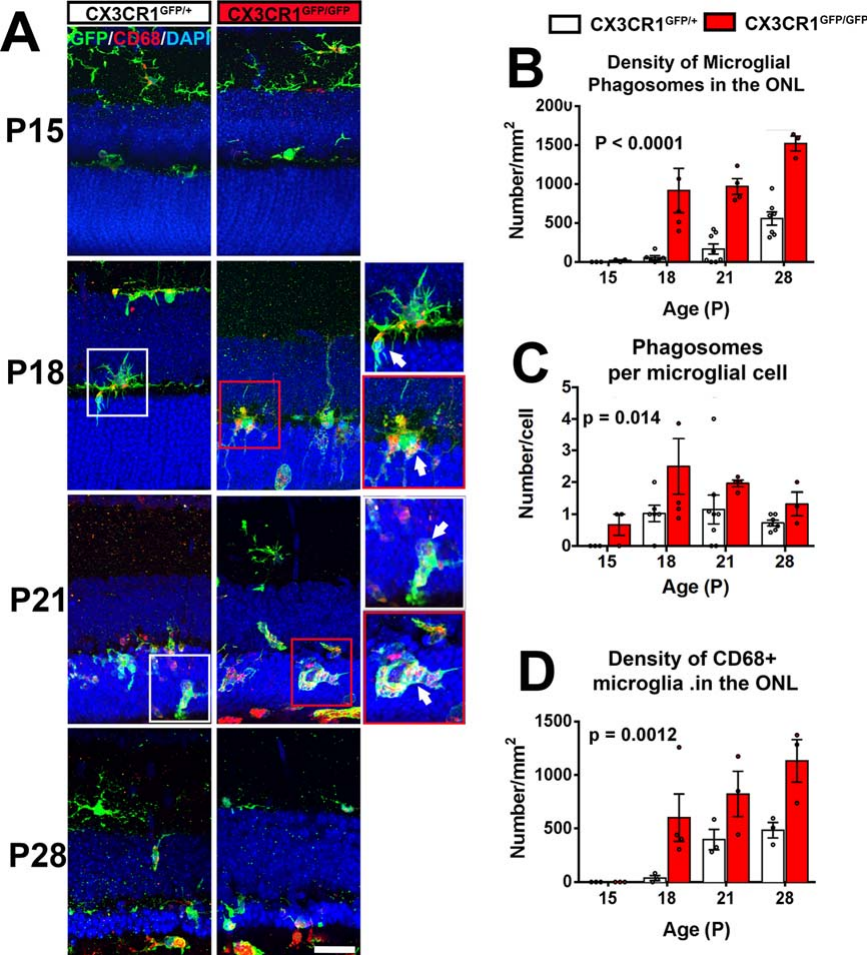
CX3CR1‐deficient microglia demonstrates increased phagocytosis of photoreceptors in the rd10 retina. (**A**) Comparison of retinal sections from rd10;CX3CR1^GFP/+^ and rd10;CX3CR1^GFP/GFP^ mice at different postnatal (P) ages. Panels show increased CD68 immunopositivity (*red*) in infiltrating microglia in rd10;CX3CR1^GFP/GFP^ retina which contained a greater number of intracellular phagosomes relative to the rd10;CX3CR1^GFP/+^ retina (*inset, arrows*). Scale bar, 30 μm. The density of phagosomes in ONL microglia (**B**), the mean number of phagosomes per microglial cell (**C**), and the number of activated CD68+ microglia in the ONL (**D**), were all generally increased in rd10;CX3CR1^GFP/GFP^ retinas relative to rd10;CX3CR1^GFP/+^ retinas. Column heights (in B, C, D) indicate mean, error bars indicate ±SEM. (*N* = 3–8 animals of each genotype and age, p values indicate comparisons between genotypes across all time‐points, two‐way ANOVA.). [Color figure can be viewed in the online issue, which is available at wileyonlinelibrary.com.]

We had discovered previously that retinal microglia contribute to photoreceptor demise in different RP models by the primary phagocytosis of living photoreceptors and the increased production of inflammatory cytokines, particularly IL1β (Zhao et al., 2015). In the current study, we investigated if these mechanisms underlie CX3CR1‐mediated differences in photoreceptor degeneration. To investigate microglial phagocytosis activity *in vivo*, we performed morphological analysis of phagocytic microglia in rd10;CX3CR1^GFP/+^ and rd10;CX3CR1^GFP/GFP^ animals. We found that CX3CR1‐deficient microglia were significantly enriched in phagosomes containing photoreceptor nuclei (Fig. 2A). The total density of microglial phagosomes in the ONL (*P* < 0.0001, two‐way ANOVA) and phagosome number per microglia (*P* = 0.014, two‐way ANOVA) were significantly increased in the rd10;CX3CR1^GFP/GFP^ retina (Fig. 2B,C). In addition, the number of infiltrating microglia immunopositive for CD68, a lysosome‐associated membrane protein (LAMP) and scavenger receptor, was also significantly higher in rd10;CX3CR1^GFP/GFP^ retina (*P* = 0.0012, two‐way ANOVA)(Fig. 2D). These indicated that CX3CR1‐deficient microglia in the rd10 retina demonstrated greater phagocytic activity and were engaged in increased photoreceptor phagocytosis relative to CX3CR1‐sufficient microglia during degeneration.

**Figure 3:**
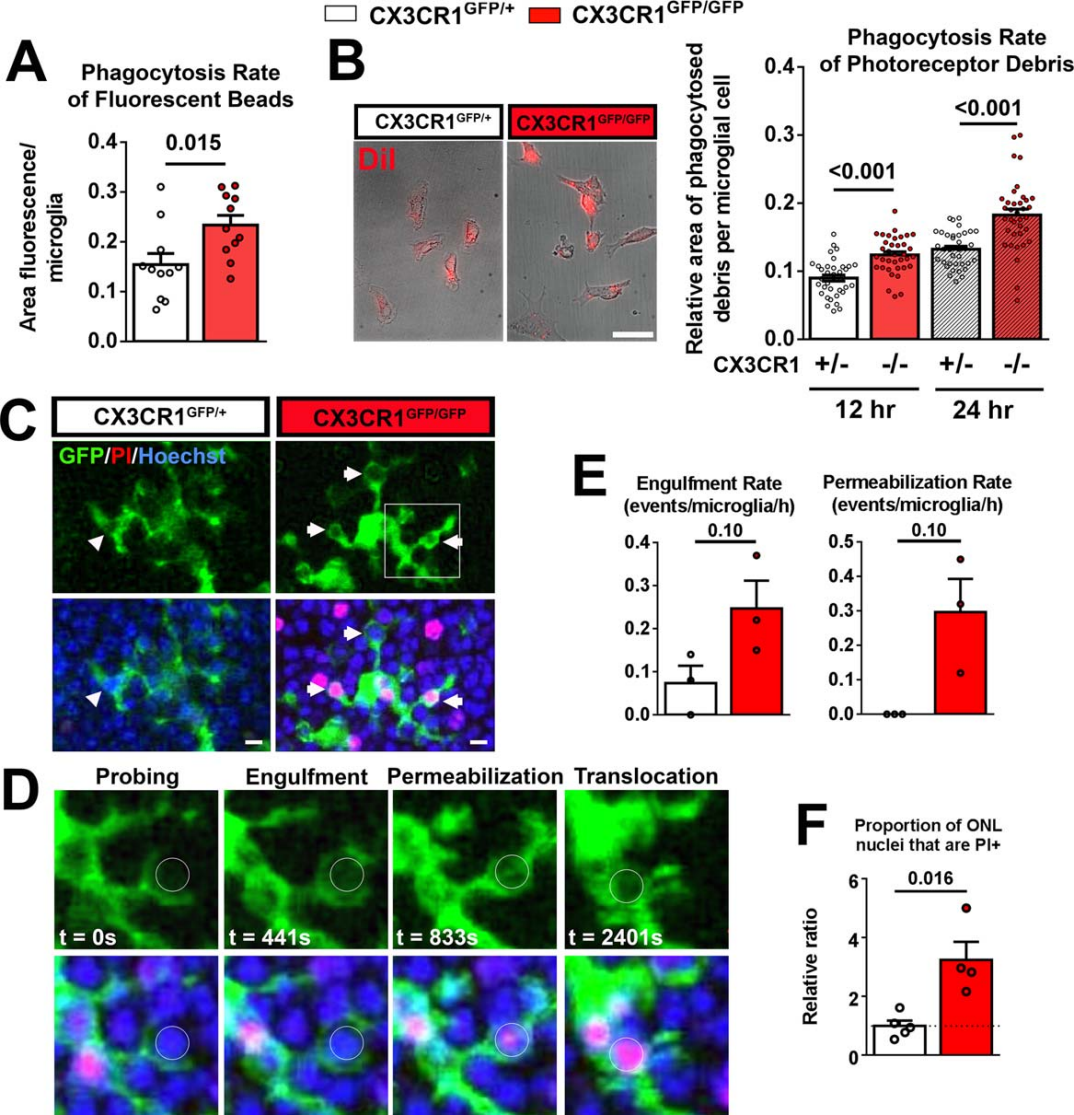
CX3CR1‐deficiency in retinal microglia is associated with increased phagocytic activity. Phagocytosis in microglia cultured from the retinas of CX3CR1^GFP/+^ and CX3CR1^GFP/GFP^ mice were evaluated in two *in vitro* phagocytosis assays. (**A**) Microglia of each genotype was incubated *in vitro* with fluorescently labeled beads for 2 h and the internalization of beads by phagocytosis was scored. CX3CR1^GFP/GFP^ microglia demonstrated a significantly higher rate of phagocytosis relative to CX3CR1^GFP/+^ microglia (*n* = 11 replicates for each genotype, *P* value from two‐tailed unpaired *t* test with Welch's correction). (**B**) Cultured microglia of each genotype were incubated with cellular debris from DiI‐labeled 661W photoreceptor cells for 12 and 24 h and mean internalization of DiI+ material by microglia (*left panels*) were measured. Scale bar, 5 μm. Significantly, more phagocytosis of photoreceptor debris was demonstrated by CX3CR1^GFP/GFP^ microglia relative to CX3CR1^GFP/+^ microglia (*N* = 36 imaging fields from six biological replicates for each genotype, *P* values from two‐tailed multiple unpaired t tests, corrected for multiple comparisons, Holm–Sidak method). (**C**) Microglial phagocytosis of photoreceptor nuclei in the ONL was monitored by live imaging in retinal explants from rd10;CX3CR1^GFP/+^ and rd10;CX3CR1^GFP/GFP^ mice isolated at P22‐24. Photoreceptor nuclei and permeabilized cells were labeled with Hoechst (*blue*) and propidium iodide (PI, *red*). Morphologically, rd10;CX3CR1^GFP/GFP^ microglia contained more phagosomes (*arrowheads*) than rd10;CX3CR1^GFP/+^ microglia. Scale bar, 5 μm. (**D**) Magnified time‐lapse views from the inset in (C) demonstrate the sequential steps in the phagocytic process (circle indicates the nucleus of a phagocytosed photoreceptor) that involve: (1) initial probing of target cell, (2) engulfment of target cell by enveloping microglial processes, (3) permeabilization of the cell membrane of the target cell as evidenced by the development of PI‐labeling (*red*), and (4) translocation of photoreceptor nucleus toward the microglial soma. (**E**) The rates of engulfment and permeabilization events were scored and compared between the two genotypes of microglia. These mean rates tended to be greater for CX3CR1^GFP/GFP^ relative to CX3CR1^GFP/+^ microglia but did not reach statistical significance (*N* = 3 recordings from each genotype, *P* values from Mann–Whitney test). (**F**) The relative proportion of ONL nuclei that were PI‐positive in each imaging field was compared between genotypes and normalized to that in rd10;CX3CR1^GFP/+^ retina. The proportion of permeabilized ONL nuclei was also significantly greater than in rd10;CX3CR1^GFP/GFP^ retinas than in rd10;CX3CR1^GFP/+^ retinas (*N* = 4–5 recordings from each genotype, *P* values from Mann–Whitney test). Column heights (in A, B, E, F) indicate mean, error bars indicate ±SEM. [Color figure can be viewed in the online issue, which is available at wileyonlinelibrary.com.]

To directly assess the effect of CX3CR1 deficiency on microglial phagocytosis activity, we cultured microglia from the retinas of CX3CR1^GFP/+^ and CX3CR1^GFP/GFP^ mice and assessed them in two separate *in vitro* phagocytosis assays. We found that CX3CR1^GFP/GFP^ microglia demonstrated significantly greater *in vitro* phagocytosis of fluorescent beads relative to CX3CR1^GFP/+^ microglia (Fig. 3A). Similarly, CX3CR1^GFP/GFP^ microglia demonstrated greater internalization of cellular debris from 661W photoreceptor cells (Fig. 3B). To further investigate how CX3CR1^GFP/GFP^ microglia are altered in their interaction with photoreceptors *in situ*, we performed live cell *ex vivo* imaging of microglial phagocytosis in retinal explants from rd10;CX3CR1^GFP/GFP^ and rd10;CX3CR1^GFP/+^ animals. Retinal microglia could be visualized as a result of their GFP expression, while photoreceptor nuclei labeled by Hoechst staining. Propidium iodide (PI), a DNA‐binding fluorescent marker which is nonpermeant to living cells but labels nucleic acids in the nuclei of permeabilized dying cells, was added to the surrounding medium. We observed that in both genotypes, infiltrating microglia interacted with photoreceptor nuclei via repeated contacts made by dynamically motile processes (Fig. 3C,D, Supporting Information Movie). As previously described (Zhao et al., 2015), these transient contacts occasionally culminated in overt phagocytosis in which the contacting microglial process engulfed the photoreceptor soma completely, enclosing it in a phagosome. The photoreceptor‐containing phagosomes were subsequently translocated within the microglial cytoplasm in the direction of the microglial soma. Following engulfment, the nucleus of the phagocytosed photoreceptor was at times observed to develop PI‐labeling within the phagosome, indicating membrane permeabilization of the engulfed photoreceptor following phagocytosis. Comparisons between the dynamic phagocytic activity of microglia of the two genotypes revealed that CX3CR1‐deficient microglia in demonstrated higher rates of photoreceptor engulfment and permeabilization of engulfed photoreceptors (both measured as events per microglia per hour) relative to CX3CR1‐sufficient microglia (Fig. 3E). A survey of photoreceptor nuclei in the ONL also demonstrated a greater prevalence of PI‐positive nuclei in the ONL of rd10;CX3CR1^GFP/GFP^ vs. rd10;CX3CR1^GFP/+^ retinas. Taken together, these findings show that CX3CR1‐deficiency in retinal microglia is correlated with greater phagocytic activity in the form of higher rates of photoreceptor phagocytosis and intra‐lysosomal breakdown, which can provide a mechanism contributing to accelerated disease progression in the rd10 retina.

### CX3CR1 Deficiency is Associated with Increased Microglial Activation in the rd10 Retina

**Figure 4:**
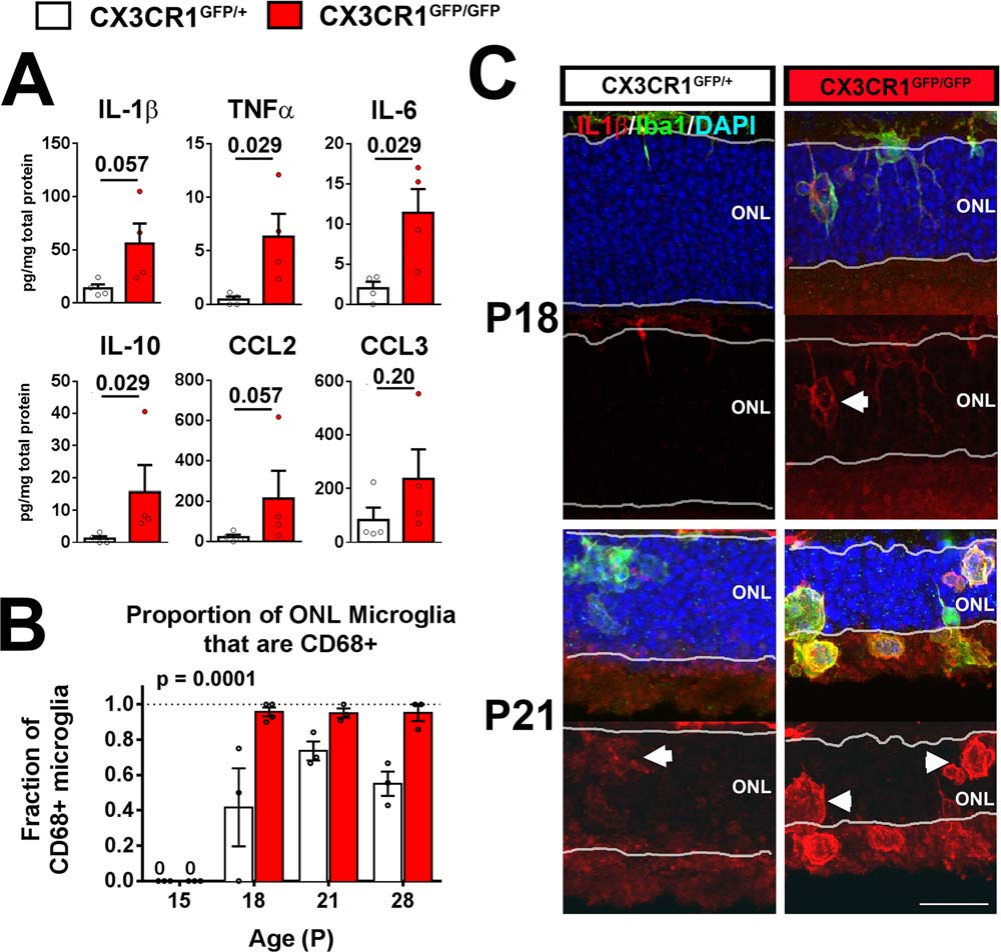
CX3CR1 deficiency in retinal microglia results in increased expression of proinflammatory cytokines in the rd10 retina. (**A**) Protein levels of cytokines in the retinas of rd10;CX3CR1^GFP/+^ and rd10;CX3CR1^GFP/GFP^ mice at P21 were measured and compared. Levels of cytokines were generally increased in rd10;CX3CR1^GFP/GFP^ retinas (*N* = 4 biological replicates from each genotype, *P* values from Mann–Whitney test). (**B**) The relative proportion of GFP‐expressing microglia were also immunopositive for the activation marker CD68 was measured in the ONL of rd10;CX3CR1^GFP/+^ and rd10;CX3CR1^GFP/GFP^ retinas at ages P15, P18, P21, and P28. A significantly greater fraction of infiltrating microglia in rd10;CX3CR1^GFP/GFP^ retinas demonstrated CD68+ positivity, demonstrating a generally increased activation status in infiltrating microglia. Column heights (in A, B) indicate mean, error bars indicate ±SEM, individual data points represent data from a single experimental animal (*N* = 3–4 animals of each genotype and age, *P* values indicate comparisons between genotypes across all time‐points, two‐way ANOVA) (**C**) Immunohistochemistry analysis demonstrate increased IL IL‐1β (*red*) immunopositivity in rd10;CX3CR1^GFP/GFP^ retina at both P18 and P21 time points that were colocalized with Iba1+ infiltrating microglia (*green, arrowheads*) in the ONL. Scale bar, 25 μm. [Color figure can be viewed in the online issue, which is available at wileyonlinelibrary.com.]

In addition to increased microglial phagocytosis, another mechanism by which CX3CR1‐deficient microglia can potentiate photoreceptor degeneration is via the increased production of inflammatory cytokines. Elevated levels of inflammatory cytokines in the outer retina have been previously linked with increased photoreceptor death in models of retinal degeneration(Kohno et al., 2013, 2014). We found that protein levels of inflammatory cytokines, typical of those secreted by activated microglia, are generally elevated in rd10;CX3CR1^GFP/GFP^ compared with rd10;CX3CR1^GFP/+^ retina (Fig. 4A). These likely arise from not only from the increased numbers of microglia infiltrating in ONL of the rd10;CX3CR1^GFP/GFP^ retina but also from the increased activation of individual microglia (CX3CR1^GFP/GFP^ vs. CX3CR1^GFP/+)^ . We found that the proportion of activated CD68‐immunopositive microglia was significantly higher in rd10;CX3CR1^GFP/GFP^ retina across the time of degeneration (*P* = 0.0001 across all time‐points, two‐way ANOVA) (Fig. 4B). Also, microglial‐associated immunopositivity for IL1β, a cytokine linked with increased microglial photoreceptor neurotoxicity(Hu et al., 2015; Zhao et al., 2015), was increased in rd10;CX3CR1^GFP/GFP^ vs. rd10;CX3CR1^GFP/+^ microglia in the ONL (Fig. 4C). These findings indicate that the increased inflammatory milieu induced by CX3CR1‐deficient microglia may additionally contribute to accelerated photoreceptor degeneration in rd10;CX3CR1^GFP/GFP^ retina.

### Increased CX3CL1‐CX3CR1 Signaling Modulates Photoreceptor Loss in the rd10 Retina

**Figure 5:**
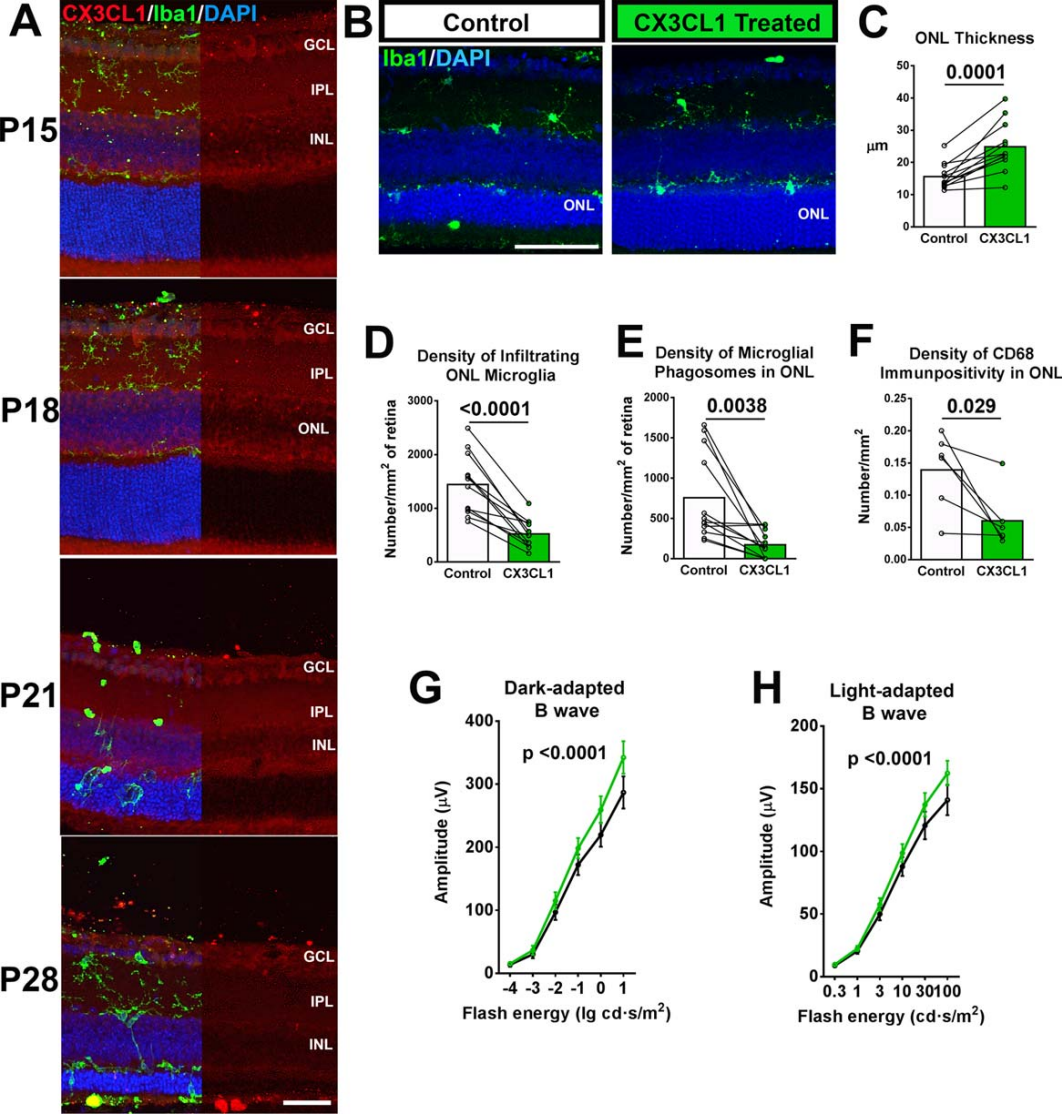
Increased CX3CL1‐CX3CR1 signaling in retinal microglia induced by exogenous delivery of CX3CL1 ameliorates photoreceptor degeneration in rd10 mice. (**A**) CX3CL1, the ligand for CX3CR1, is endogenously expressed in the inner retina across the period of rod degeneration (P15‐P28) in the rd10 retina. Immunohistochemistry analysis demonstrated immunopositivity for CX3CL1 in the ganglion cell layer (GCL), the inner plexiform layer (IPL), and the inner and outer borders of the inner nuclear layer (INL) from P15 to P21. Immunopositivity decreased across the retina at P28 with some residual expression in the GCL. Scale bar, 30 μm. (**B**–**H**) Exogenous CX3CL1 (66–100 ng in injection volume of 1 μL) was delivered by intravitreal injection to one eye (i.e., the treated eye) of P20 CX3CR1^GFP/+^ mice, the contralateral eye (i.e., the control eye) was given the same dose of CX3CL1 that was previously inactivated with heat treatment. Treated animals were assessed post‐treatment at P26 by histology and electroretinography (ERG). Retinal sections from equivalent regions of the control and treated eyes of a representative P26 animal are shown in (B), Scale bar, 50 μm. (C) Comparisons of ONL thickness show decreased photoreceptor degeneration in CX3CL1‐treated eyes. These changes were correlated with decreased numbers of infiltrating microglia (D) and microglial phagosomes (E) in the ONL (*n* = 12 animals). There were also fewer activated CD68+ microglia among infiltrating cells in the ONL (F) (*N* = 6 animals). (*P* values from C–H from paired *t* tests). (G, H) Electroretinographic (ERG) recordings demonstrated significantly increased *b*‐wave amplitudes in dark‐ (*left*) and light‐adapted (*right*) responses in treated vs. control eyes (*n* = 17 animals, *P* values from two‐way ANOVA). Column heights (in C–F) indicate mean, error bars (in C–H) indicate ±SEM, individual data points indicate a single experimental animal, with connecting lines indicating paired eyes of the same animal. [Color figure can be viewed in the online issue, which is available at wileyonlinelibrary.com.]

The above implication of CX3CR1 in regulating microglia‐mediated photoreceptor degeneration highlights a potential therapeutic strategy to slow down degeneration via the modulation of CX3CL1‐CX3CR1 signaling. As CX3CL1 is expressed broadly in the retina during rod degeneration in the rd10 retina (Fig. 5A), we explored whether exogenous CX3CL1 delivered to the rd10 retina can further increase CX3CL1‐CX3CR1 signaling in microglia beyond endogenous levels and decrease microglia neurotoxicity. We injected rd10;CX3CR1^GFP/+^ mice with recombinant CX3CL1 protein in one eye (i.e., the treated eye) at P20 prior to the onset of degeneration; the contralateral control eye was injected with an equivalent dose previously heat‐inactivated CX3CL1. We found through pair‐wise comparisons at P26 that the treated eye demonstrated a significantly slowed the rate of photoreceptor degeneration as evidenced by greater preservation of ONL thickness in treated vs. control eyes (Fig. 5B,C). Treated eyes relative to control eyes also demonstrated (1) a decreased density of microglia infiltrating the ONL (Fig. 5D), (2) a decreased density of microglial phagosomes (Fig. 5C), and (3) a decreased density of CD68‐immunpositive microglia in the ONL (Fig. 5F), indicating that upregulation of CX3CL1‐CX3CR1 signaling in microglia decreased microglial phagocytosis and activation, phenotypes converse of those observed in CX3CR1‐deficient microglia. Consistent with these findings, electroretinographic evaluation demonstrated slightly but significantly greater preservation of dark‐adapted and light‐adapted b‐wave amplitudes at P26 in treated eyes vs. control eyes (Fig. 5G,H). Taken together, these findings indicate that upregulation of CX3CL1‐CX3CR1 signaling decreased microglial phagocytosis and activation, resulting in greater morphological and functional preservation of photoreceptors in the rd10 retina.

## Discussion

While the primary etiology of photoreceptor degeneration in inherited retinal degenerations originate predominantly from mutations in photoreceptor‐expressed genes, the non‐cell‐autonomous contributions that microglia make to the overall rate and extent of photoreceptor degeneration have been increasingly appreciated. Activation and recruitment of microglia are known to occur concurrently with photoreceptor degeneration in RP animal models (Roque et al., 1996; Zeiss and Johnson, 2004) and human histopathological specimens (Gupta et al., 2003; Zhao et al., 2015). More than merely acting as “bystanders” to ongoing photoreceptor degeneration, microglia have been demonstrated to contribute directly; interventions that suppress microglial activation (Peng et al., 2014), cytokine signaling (Zhao et al., 2015), pro‐oxidative properties (Zeng et al., 2014), and phagocytosis (Zhao et al., 2015), can successfully ameliorate photoreceptor degeneration. In the current study, we documented the effect of genetic deficiency of CX3CR1, a gene specifically expressed in retinal microglia, on the severity of photoreceptor degeneration. This further implicates the causal involvement of microglia and connects their physiological state to the overall rate of disease progression, lending credence to the strategy of reducing non‐cell autonomous contributions of microglia to photoreceptor degeneration as a means of slowing down visual loss in diverse genetic etiologies of RP (Karlstetter et al., 2015).

CX3CL1‐CX3CR1 signaling appears to be a central mechanism in neuron‐to‐microglia communication in the CNS. Constitutively expressed by neurons (Harrison et al., 1998), the ligand CX3CL1 exerts effects on multiple aspects of microglial physiology, including actions on neurons and synapses in the developing and adult brain (Paolicelli et al., 2014). During development, CX3CL1‐CX3CR1 signaling influences microglial regulation of neuronal survival (Sierra et al., 2010; Ueno et al., 2013), and synaptic maturation and refinement (Hoshiko et al., 2012; Paolicelli et al., 2011) in the brain. During adulthood, CX3CL1‐CX3CR1 signaling regulates microglial interactions with cortical synapses, modulating synaptic transmission (Maggi et al., 2011; Ragozzino et al., 2006) in ways that affect learning and memory (Rogers et al., 2011). In brain neurodegenerative pathologies, CX3CL1‐CX3CR1 signaling is important in determining disease outcomes in models of Parkinson's disease (Cardona et al., 2006), multiple sclerosis (Garcia et al., 2013), and Alzheimer's disease (Cho et al., 2011; Fuhrmann et al., 2010).

In the retina, CX3CL1‐CX3CR1 signaling is similarly prevalent as in the rest of the CNS, as evidenced by maintained expression of CX3CR1 expression in retinal microglia (Combadiere et al., 2007), and the constitutive expression of CX3CL1 in retinal neurons (Silverman et al., 2003; Zieger et al., 2014). In the uninjured adult retina, while the ablation of CX3CL1‐CX3CR1 signaling does not culminate in gross changes in retinal morphology (Luhmann et al., 2012; Vessey et al., 2012), it induces a slight but significant slowing of constitutive microglial process dynamics (Liang et al., 2009) which, given the role of microglia in synaptic maintenance, are likely related to alterations in the dendritic structure and function of inner retinal neurons (Vessey et al., 2012). Retinal levels of CX3CL1 appear sensitive to pathological perturbations, and have been reported to be upregulated in response to inflammatory mediators (Silverman et al., 2003)and light injury (Zhang et al., 2012). Interestingly, in the rd10 retina, CX3CL1 expression, while detectable across the time of photoreceptor degeneration, was altered in isoform composition and actually decreased at the mRNA and protein levels relative to wild type controls (Zieger et al., 2014). As such, CX3CL1 expression may be regulated in a complex manner in settings of neural degeneration, and may involve post‐translational changes that modify the biological activity of the chemokine. Further study into the regulation of CX3CL1 expression may be insightful in uncovering homeostatic mechanisms in CX3CL1‐CX3CR1 signaling. Maintenance of appropriate levels of signaling may be pathogenically significant, as decreased CX3CR1 signaling has been related to worsened outcomes and increased microglial activation in a number of retinal disease models (Chen et al., 2013; Dagkalis et al., 2009; Kezic et al., 2013; Peng et al., 2014; Sennlaub et al., 2013; Wang et al., 2014; Zhang et al.,, 2012). These observations together highlight CX3CL1‐CX3CR1 signaling as an influential mode of communication active during physiological and pathological conditions in the retina and across in the CNS.

In the current study, we have found that a key mechanism underlying the effect of CX3CR1‐mediated signaling on photoreceptor degeneration is through the regulation of microglial phagocytosis of photoreceptors. CX3CR1 signaling is known to regulate microglial phagocytosis in other contexts, such as synaptic phagocytosis during development (Milior et al., 2015; Paolicelli et al., 2011), and amyloid plaque clearance in Alzheimer's disease models (Lee et al., 2010; Liu et al., 2010). However, how CX3CL1‐CX3CR1 signaling regulates primary microglial phagocytosis of living rods in photoreceptor degeneration has not been previously explored. In our previous work (Zhao et al., 2015), we have described the dynamic migration of microglia to the outer retina early in the course of rd10 retinal degeneration and their extensive interaction and physical contact with photoreceptors via motile microglial processes which eventually culminates in the overt phagocytosis of nonapoptotic rods. We demonstrate here that multiple steps in the overall process involving microglial migration into the ONL, dynamic contact with photoreceptors, and primary phagocytosis of nonapoptotic rods, were all significantly augmented in the absence of CX3CR1 signaling. This altered phagocytic physiology of CX3CR1‐deficient microglia could have arisen from abnormal microglial development; however, as CX3CR1‐deficient microglia in the adult retina are normal in their lamina distribution and density, and demonstrate only subtle changes in the dendritic morphology (Liang et al., 2009; Vessey et al., 2012), it is more likely that constitutive CX3CL1‐CX3CR1 signaling in the retina serves to negatively regulate and limit the extent of microglial phagocytic behavior and activity. The presence of this regulatory effect is supported by our observations here that exogenous CX3CL1 administration can decrease phagocytic activity and slow down structural and functional loss in the rd10 retina.

Additionally, CX3CL1‐CX3CR1 signaling is also likely to limit microglial contribution to photoreceptor degeneration by downregulating microglial activation and decreasing production of inflammatory cytokines. Previous work have demonstrated that in addition to primary phagocytic clearance, retinal microglia can potentiate photoreceptor death by increasing the rate of photoreceptor apoptosis via proinflammatory cytokines, particularly IL1β (Hu et al., 2015; Zhao et al., 2015). Our observations that CX3CR1‐deficient microglia in the rd10 retina demonstrated increased activation markers and higher expression of IL1β indicate that constitutive CX3CR1 signaling serves to repress microglial activation and cytokine production (Biber et al., 2007) and negatively regulate the neurotoxic capabilities of microglia.

The elucidation of the cellular and molecular mechanisms governing microglial contributions to photoreceptor degeneration presents opportunities for targeting microglia as a therapeutic approach in RP. Our findings here regarding the protection conferred by intraocular CX3CL1 delivery highlight the possibility that modulation of CX3CL1‐CX3CR1 signaling, like in other CNS pathologies (D'Haese et al., 2012), may have therapeutic potential. This approach holds promise as multiple genetic etiologies of human RP and animal models of the disease appear to share the involvement of microglial phagocytosis as a common factor (Zhao et al., 2015), indicating a potential broad application. However, as induced increases in CX3CL1‐CX3CR1 signaling have also been associated with deleterious consequences in particular models of neural degeneration depending on the context (Limatola and Ransohoff, 2014), general application of this approach requires further testing and confirmation across different dose ranges and disease stages.

While CX3CR1, in being specifically expressed in microglia in the retina, is unlikely to directly influence phagocytosis and cytokine production in other retinal cell types, such as retinal pigment epithelial (RPE) cells, these processes may be influenced by microglial dysregulation and contribute to photoreceptor degeneration. In the healthy retina, RPE cells are principally involved in the constitutive phagocytosis of outer segments (Strauss, 2005) and the maintenance of the immune environment in the outer retina (Detrick and Hooks, 2010). Dyregulated or dysfunctional RPE phagocytosis and RPE regulation of inflammation have been linked to photoreceptor degeneration (Ferrington et al., 2016), and as such, cross‐over effects, if any, need to be taken into account in the consideration of interventions aimed at modulating microglial phagocytosis and cytokine production.

In summary, we demonstrate here that mechanisms that govern microglial contributions to photoreceptor degeneration in the rd10 retina, namely primary microglial phagocytosis of photoreceptors and inflammatory cytokine production, are negatively regulated by constitutive CX3CL1‐CX3CR1 signaling. Our findings here highlight an opportunity for translational study into the modulation of microglia as a therapeutic strategy to RP treatment.

## Supporting information

Supporting Information
